# Nanocarriers for Diagnosis and Targeting of Breast Cancer

**DOI:** 10.1155/2013/960821

**Published:** 2013-06-24

**Authors:** Arun Sharma, Nitin Jain, Rashmi Sareen

**Affiliations:** Department of Pharmaceutics, School of Pharmaceutical Sciences, Shoolini University, Bajhol, Solan, Himachal Pradesh 173229, India

## Abstract

Breast cancer nanotherapeutics is consistently progressing and being used to remove the various limitations of conventional method available for the diagnosis and treatment of breast cancer. Nanoparticles provide an interdisciplinary area for research in imaging, diagnosis, and targeting of breast cancer. With advanced physicochemical properties and better bioavailability, they show prolonged blood circulation with efficient tumor targeting. Passive targeting mechanisms by using leaky vasculature, tumor microenvironment, or direct local application and active targeting approaches using receptor antibody, amplification in the ability of nanoparticles to target specific tumor can be achieved. Nanoparticles are able to reduce cytotoxic effect of the active anticancer drugs by increasing cancer cell targeting in comparison to conventional formulations. Various nanoparticles-based formulations are in the preclinical and clinical stages of development; among them, polymeric drug micelles, liposomes, dendrimer, carbon nanotubes, and nanorods are the most common. In this review, we have discussed the role of nanoparticles with respect to oncology, by particularly focusing on the breast cancer and various nanodelivery systems used for targeting action.

## 1. Introduction

The development and inventions of various nanoscale technologies have provided new field of research among chemistry, biology, toxicology, medicine, material science, engineering, and mathematics. Nanotechnology is the manipulation of cellular and molecular components of matter. Nanotechnology yields the incredibly small particles of size ranging between tens to hundred nanometers. These small particles are known as nanoparticles, which are considered as the engineered materials mainly cluster of molecules, atoms, and molecular fragments. These innovations are referred to as nanomedicines by the National Institute of Health and have the potential of carrying chemotherapeutic agents to the targeted site. Being a nanotechnologically engineered material, nanocarriers must have four characteristics of its own such as size of the material should be in nanometer, properties of the material should be in nanoscopic dimensions, behavior of the material should be displayable with suitable mathematical expression, material should be man-made [1]. Rationale behind the development on nanocarriers is that polymeric particles, metal, and semiconductors have unique structural, magnetic, optical, and electronic properties which make them a suitable drug delivery carrier for targeting [2].

**Figure 1:**
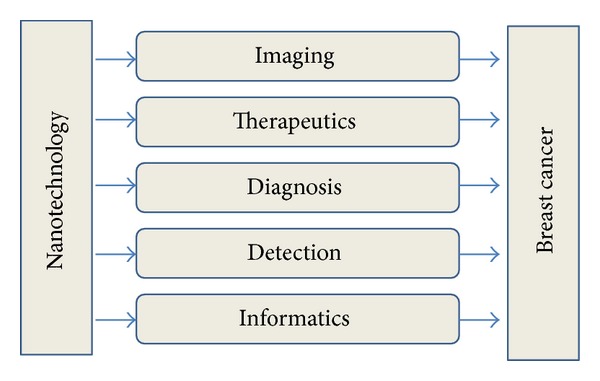
Area of nanotechnology in breast cancer.

Nanoscale devices form the concept of biodegradable self-assembled nanoparticles which can be targeted to the cancer-affected area and can be used as contrast imaging agents [3]. Breast cancer is a major ongoing public health problem, and at present, there are less curative options for the patients suffering from breast cancer, while emerging nanotechnologies give a promising new approach for the early detection and treatment of breast cancer. Nanoparticles provide an interdisciplinary area for research in imaging, diagnosis and targeting of breast cancer (Figure 1).

## 2. Human Breast Cancer

It is also called prostate cancer which originates from breast tissue. Breast cancer is most frequently diagnosed in women; approximately up to 7% of breast cancers are being diagnosed in women having their age below 40 years and less than 4% in women below the age of 35 years [4]. In young women, the breast cancer is uncommon [5]. Breast cancer is a heterogeneous disease and has different subtypes, which are based on the expression level of progesterone receptor, estrogen receptor, and HER-2/neu receptor (human epidermal growth factor receptor 2) [6]. Breast cancer stem cells play a major role in growth and formation of metastastic breast cancer. Breast cancer stem cells have a potential for undergoing self-renewal and side by side give rise to daughter cells which results in the formation of tumor cells in bulk having self-replicating potential. Breast cancer stem cells make small-small part of most tumors, whereas in others like in melanoma it comprises 25% of total mass [7]. Based on the TNM (tumor nodes metastasized) system, breast cancer can be divided into four stages: based on the size of tumor (T), whether the tumor has spread to the lymph nodes (N) in the armpits or not, and whether the tumor has metastasized (M).

Types of stages in breast cancer are as follows.

1. Stage 0: it consists of three types of breast carcinoma.
  - Ductal carcinoma *in situ* (DCIS): this condition is noninvasive and the abnormal cells are found in lining of the breast duct, but the spreading of the abnormal cells is not outside the tissues of breast.
  - Lobular carcinoma *in situ* (LCIS): in this condition, the abnormal cells are present in the lobules of the breast. This rarely occurs as invasive cancer. Presence of abnormal cells in lobules increases the risk of breast cancer.
  - Paget disease of the nipple: in this condition, abnormal cells are found in nipple only.
2. Stage I: it is divided into two stages Ia and Ib.
  - Stage Ia: tumor is 2 cm or small and not found outside the breast.
  - Stage Ib: small clusters are found in the lymph nodes and either tumor is 2 centimeters or not found in breast.
3. Stage II: it is divided into Stage IIa and Stage IIb.
  - Stage IIa: tumor is found to be larger than 2 cm but not larger than 5 cm. Cancer has not spread to the lymph nodes.
  - Stage IIb: tumor is larger than 2 cm but not greater than 5 cm. Cancer spreads to 1 to 3 axillary lymph nodes or to the lymph node near the breastbone.
4. Stage III: it is divided into IIIa and IIIb.
  - Stage IIIa: the tumor is larger than 5 cm and cancer spreads to 1 to 3 axillary lymph nodes.
  - Stage IIIb: the tumor spreads to 9 axillary lymph nodes.
  - Stage IIIc: tumor may be of any size causing swelling or ulcer and has spread to chest wall. Cancer has spread to 10 or more axillary lymph nodes.

In treatment point of view, Stage IIIc is divided into operable and inoperable.

(5) Stage IV: cancer has spread to other parts of the body, mostly to lungs, bone, or liver. 

Now nanotechnology comes up with a bright way to overcome the problem related to breast cancer. Many researchers focus on different types of nanotechnology-based drug delivery system and their mechanism of action in such type of carcinoma. Various types of nanoparticles are used for the detection of breast carcinoma; among these, carbon nanorods (gold nanorods [8]), nanowires (Au nanowires [9]), and nanobarcodes are the most common. Semiconductor quantum dots (QDs) are a new advancement in nanotechnology; these are small nanoscale light-emitting particles and are better in comparison to fluorescent protein and organic dyes. Unique electronic and optical properties of semiconductor quantum dots make them suitable agents for cellular and *in vivo* biomolecular imaging [10]. Yu et al. had synthesized cadmium oxide-selenium powdered QD of 2-nanometer diameter approximately that produces a blue emission and a 7-nanometer diameter quantum dots showing red light emission [11]. Superimposed optical images and X-ray have shown high resolution and high sensitivity for the location of not only bigger breast cancer but also for small abnormal tumor daughter cells. Chemotherapy in the form of nanoparticles can be delivered by active and passive pathway. Nanotechnology is used in molecular cancer diagnosis by employing biomarker and nanoparticles probes. Multiple ligands can be conjugated on tiny single nanoparticles and provide a multivalent effect for increased specificity and binding affinity, hence used as suitable diagnostic agent.

## 3. Chemotherapeutic Nanoparticles

Chemotherapeutic drugs are “cytotoxic” in nature, which means cell-killing drugs. They play a vital role in the management and treatment of both initial-stage breast cancer and advanced breast cancer. Cytotoxic chemotherapy is essential for palliation of women with hormone-insensitive or hormone-refractory breast cancer and is administered into human body by taking therapeutic goals into consideration such as relief from pain, disease progression, relief from symptoms, prolonged life of patient, and improvement in mood disturbances of suffering women [12]. It is administered orally or by intravenous injection. It works systemically by killing cancer cells throughout the body along with normal cells, which leads to various short-term and long-term side effects. Mostly chemotherapy is used in advanced breast cancer, but may also be used to treat early-stage breast cancer. By using nanoparticles as carrier, cytotoxic side effects may be reduced and targeting may be achieved.

Even the most advanced chemotherapeutic agents do not differentiate between normal cells and cancerous cells efficiently, which leads to nonspecific distribution of drug in the body and causes systemic toxicity and adverse effects [13]. The maximum allowable dose of the drug gets limited; in order to achieve the desired therapeutic effect in the tumor tissue, large quantity of drug has to be administered in order to achieve anticancer effect, but this is not economical and undesirable toxicity may also appear [13, 14]. Nanoparticles are the promising carrier system for the targeted delivery of chemotherapeutic agents by using both active and passive targetings, and systemic toxicity or normal cell toxicity can be avoided [14].

## 4. Active and Passive Drug Delivery

**Figure 2:**
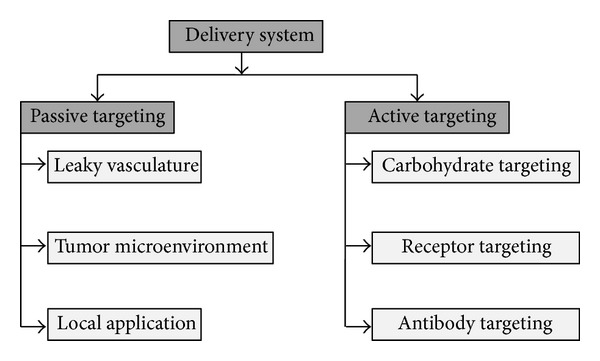
Types of targeting by nanodelivery system.

Mostly the nanoparticles accumulate in tumor cells as expected because of pathophysiological characteristics of tumor blood supplying vessels. There is an increased demand of oxygen and nutrients to the tumor cells or tissue as it is increasing in size as well as in its shape. In order to supply nutrients and oxygen, new blood capillary system is being developed which is not developed properly and hence becomes permeable to some particles of specific size [15]. Types of targeting are shown in Figure 2.

### 4.1. Passive Targeting by Nanoparticles

**Figure 3:**
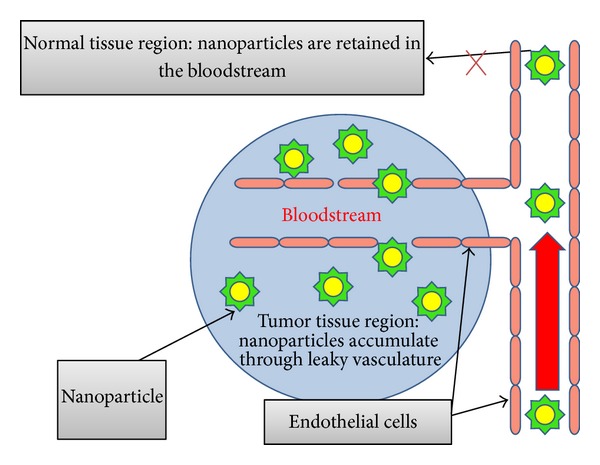
Passive targeting.

Passive targeting can differentiate between normal and tumor tissues and has the advantage of direct permeation to tumor tissue (Figure 3). Drug administered passively in the form of prodrug or inactive form, when exposed to tumor tissue, becomes highly active. Nanoparticles that are expected to show localization on specific tissues or at specific sites of disease follow the biological mechanisms such as ERS (enhanced retention system) or EPS (enhanced permeation system) effect. To prolong the circulation and to achieve increased targeting efficiency, the size should be below 100 nanometers in diameter and the surface of the nanoparticles should be hydrophilic in nature in order to circumvent clearance by macrophages. The hydrophilic surface of the nanoparticles provides protection against plasma protein adsorption to the surface, and this can be made possible by using hydrophilic polymer coating, like polyethylene glycol (PEG), polysaccharides, poloxamines, or poloxamers or by using block or branched amphiphilic copolymer [16, 17]. Passive targeting system is further classified into (a) leaky vasculature, (b) tumor microenvironment, and (c) local drug application.

*(1) Leaky Vasculature.* Maeda and Matsumura had first displayed the enhanced permeation and retention effect by using polymer to form nanoparticles. Concept of enhanced permeability and retention is based on two factors [18]. (a) The capillary endothelium system in malignant tissue shows more permeation to macromolecules in comparison to normal tissue endothelium; this makes circulating polymeric nanoparticles permeable into the tumor. (b) Tumor lacks lymphatic drainage; hence, more drug gets accumulated in side tumor tissue. By using a suitable biodegradable polymer, the concentration of drug gets 10 to 100 times higher than that of free circulating drug.

*(2) Tumor Microenvironment.* Tumor microenvironment provides the advantage of the passive drug targeting. Active state of chemotherapeutic agent is conjugated with tumor-specific material and administered into the body. When this drug-polymer conjugate reaches its desired destination, tumor environment converts into more active form. This phenomenon is called tumor activated prodrug therapy. Mansour et al. had developed an albumin-bound form of doxorubicin and showed in an *in vitro* study, that doxorubicin was efficiently cleaved by matrix metalloproteniase-2 [19].

*(3) Local Drug Application.* Direct application of the chemotherapeutic agent locally to tumor site prevents systemic toxicity and increased concentration of drug at the tumor site. Nomura et al. had synthesized intratumoral injection of mitomycin c-dextran conjugate; this results in increased concentration of anticancer drug at tumor site and decreased systemic toxicity [20]. Prabha and Labhasetwar had worked on nanoparticles-mediated wild-type p53 gene for breast cancer and observed the sustained and increased antiproliferative effect [21].

### 4.2. Active Targeting

**Figure 4:**
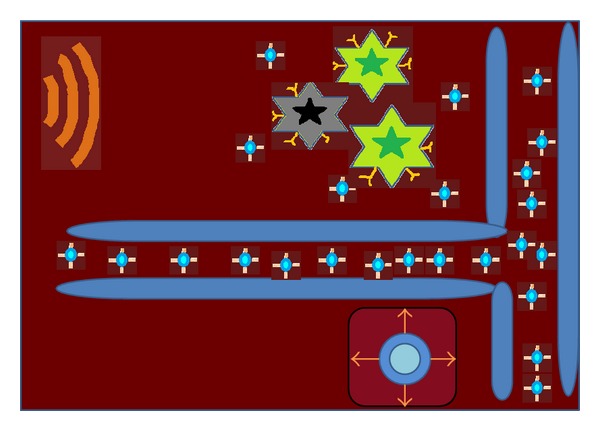
Active targeting.

By conjugating the nanoparticles with a drug to desired target site, an active targeting may be achieved (Figure 4). Active targeting allows the increased accumulation of the drug in cancer tissue. Directing the nanoparticles to the cancer cell can be done by the following ways. This approach is basically based on the specific interactions, like lectin carbohydrate, ligand receptor, and antibody-antigen [22].

*(1) Carbohydrate-Directed Targeting.* An excellent example of active drug targeting is lectin carbohydrate. Carbohydrates present on the surface of tumor cell are different than those in the normal cell. Lectin is a nonimmunological protein, which is capable of binding and recognizing the glycoproteins which are present on the surface of the cell. Certain carbohydrates interact with lectins to form the cell-specific binding moieties. These carbohydrates moieties can be used for target drug delivery system for lectins (lectin direct targeting); similarly, lectins can also be used for the targeting of the surface carbohydrates (reverse lectin targeting). Specific carbohydrate present on tumor can be targeted and anticancer effect may be achieved.

*(2) Receptor Targeting.* Endocytosis plays a major role in this type of active targeting. Ideally drug is being conjugated to polymer carrier; this carrier gets incorporated into the cell and localized at the cell surface. Once the drug-polymer conjugate reaches the tumor intracellular environment, dissociation of drug takes place and anticancer effect is being achieved.

Three essential molecules can be delivered by this targeting system:

- antigen or receptors,
- drug-polymer conjugates, and
- ligands or antibodies.

*(3) Antibody Targeting.* Kirpotin et al. had described the evidence of monoclonal antibody mechanism for targeting nanoparticles to solid tumor tissue *in vivo*. Prepared formulation was targeted towards the HER-2 (human epidermal growth factor receptor 2) cancer and was prepared by conjugating the anti-HER-2 monoclonal antibody fragments with liposomal-grafted polyethylene glycol chain. Increased cellular uptake of the drug was observed; hence, antibody targeting provides the new opportunities for drug delivery system in breast cancer [22].

## 5. Types of Nanodelivery System

Different types of nanodelivery system having different physicochemical properties with different materials have been formulated so far in order to cure diseases. Most commonly studied among these are polymeric micelles, dendrimers, liposomes, carbon nanotubes, and nanorods (Table 1).

### 5.1. Polymeric-Based Drug Carrier

The drug is either covalently bound or physically entrapped in polymer matrix, depending on the method of preparation [23]. Polymers can be divided into two groups: natural and synthetic polymers. Polymers like chitosan, albumin, and heparin occur naturally and have been a choice of material for the delivery of DNA, protein, and oligonucleotides as well as drug. Gradishar et al. had formulated conjugate of paclitaxel with serum albumin to form nanoparticles formulation. This drug-polymer conjugate has been applied for the treatment of metastatic breast [24].

Among synthetic polymers, N-(2-hydroxypropyl)-methacrylamide copolymer (HPMA), poly-1-glutamic acid (PGA), polystyrene maleic anhydride copolymer, and polyethylene glycol (PEG) are common. Polycaprolactone and polyalkyl cyanoacrylates are widely used polymers in nanodelivery. PGA is the first biodegradable polymer to be used for conjugate synthesis [25]. HPMC and PEG are nonbiodegradable synthetic polymers which are most widely used [26].

#### 5.1.1. Polymeric Micelles

Micelles are generally colloidal particles having a size range usually in between 5 and 100 nanometers in diameter. Micelles mainly consist of surface active agents (surfactant) or amphiphiles, which are made up of two different regions, hydrophobic tail and mostly hydrophilic head. Amphiphiles exist as monomers in aqueous medium at low concentration as a true solution. By increasing the concentration of amphiphiles, self-assembled aggregations are being formed called micelles within the narrow concentration window [27]. The concentration above which the micelles formation takes place is called CMC (critical micelles concentration). Above the CMC, the micelles are being formed by the dehydration of the hydrophobic tails with favorable entropy. Van der Waals bonds are responsible for the formation of micelle core by combining hydrophobic polymers in symmetrical way. Conventional oral administration of anticancer drugs showed reduced absorption and reduced bioavailability [27].

Polymeric micelles provide an excellent advantage of smaller size in comparison to liposomes. Polymer selection plays an important role in the formation of micelles, and the selection for the micelles formation is based on the characteristics of both hydrophobic and hydrophilic block polymers. Hydrophilic outer shell of the micelles gives steric stability and prevents rapid uptake of formulation by reticulo endothelial system and provides longer duration of circulation time inside the body [28]. Hydrophobic and hydrophilic polymers are the block polymers for the formation of the micelles which assemble themselves in an aqueous environment to form hydrophobic core which is being stabilized by hydrophilic shell. By arranging these block polymers, different patterns of micelles are being formed; hence, these polymers are called diblock copolymer (A-B type copolymers), triblock copolymer (A-B-C type copolymer), and grafted polymers [29].

Xue et al. had developed biodegradable diblock amphiphilic copolymer (mPEG-b-p(LA-CO-MCG) having carboxylate group for platinum chelation. The cytotoxicity of the drug-polymer conjugate towards breast cancer was lower than of cisplatin but comparable to that of oxaliplatin. This polymer conjugate showed the potential use as a targeted carrier vehicle due to its reduced side effect [30]. Zhang et al. had developed a combination of salinomycin and octreotide-modified paclitaxel-loaded PEG-B-PCL polymer micelles. This combination therapy showed improved treatment of breast cancer. Combination was designed in order to eradicate both breast cancer stem cells and breast cancer cells which cannot be eradicated by conventional chemotherapy. Elimination of cancer cell is based on the mechanism of receptor-mediated endocytosis [31]. Octreotide-modified paclitaxel follows the active targeting mechanism, whereas salinomycin follows the passive targeting mechanism. Liu et al. had formulated curcumin-loaded biodegradable self-assembled polymeric micelles called as curcumin polymeric micelles which showed good water solubility and had met the intravenous administration requirements. Sustained release and lower cytotoxicity of curcumin polymer micelles may serve as candidate for antimetastasis agent for breast cancer [32].

Polymer-based imaging with near-infrared (NIR) fluorophores provides efficient advantages for tumor imaging, such as improved plasma half-lives, large surface area, less toxicity, stability, and improved targeting. For *in vivo* imaging of tumor, NIR fluorophores are increasing its hold [33]. Along this, NIR fluorophores do not require expensive instruments, a local cyclotron, or incontinent radionuclide-labeling step [34]. Kim et al. have developed NIR Cy5.5-labeled hydrophobically modified glycol chitosan nanoparticles (HGC-Cy5.5) with molecular weight ranging from 20 to 250 kDa. *In vivo* biodistribution study revealed that low-molecular-weight HGC-Cy5.5 showed faster clearance from the body in comparison to high-molecular-weight HGC-Cy5.5, whereas high-molecular-weight HGC-Cy5.5 had high tumor targeting capacity than low-molecular-weight HGC-Cy5.5. These probes provide promising imaging agents, which are used to detect solid tumor [35]. Kim et al. have developed NIR fluorescent-activatable polymeric nanoparticles (Cy5.5) linked effector caspase-specific peptide having efficient biocompatibility and cell permeability. These nanoparticles were specifically apoptosis sensitive nanoparticles (80–100 nm). This probe could be used as an imaging agent for apoptosis in single cells [36].

#### 5.1.2. Dendrimer

Nanosized branched structures are called dendrimer. The name comes from the Greek word “dendron” which means tree-like structures. With various architectural variations, uniformity in size, branching length, shape and increased surface area can be achieved. Dendrimers show higher biocompatibility and certain changes in the structure of dendrimers; pharmacokinetic parameters can also be predictable. Hence, dendrimers can be optimal and unique carrier system for anticancer drug [37, 38]. Dendrimer can be grown towards outward direction from the central core; this process is known as divergent method designed by Newkome and Tomalia [39–41], or it may be formulated by the Frechet's method, in which the dendrimers are made toward inside direction, that is, from the periphery to inner core [42]. Dendrimers are also described on the basis of the branching unit they consist of, like dendrimer with central branch core molecule is considered as generation 0 (G0) and with each successive addition of increased branching point they may be considered as G1, G2 and so forth. Dendrimers may be categorized by terminal generation, like G6 consists of polymer with five generations of branching points. Dendrimers form the globular shape and attain higher diameter with increasing branching generation [43]. Dendrimers and dendrons are monodispersed and usually highly symmetric, spherical compounds. Dendrimer can be used as carrier system for the treatment of diseases like AIDS, cancer, malaria, and so forth.

Wang et al. had synthesized G4 polyamidoamine dendrimer (G4 PAMAM-D) conjugate with antisense oligodeoxynucleotides (ASODN). The conjugate showed more stability less toxicity, and increased bioavailability. *In vivo* studies on xenograft mice model showed that the conjugate has more accumulating efficiency to inhibit tumor vascularisation of breast tumor than naked ASODN [44]. Gupta et al. had conjugated doxorubicin (DOX) to polypropylene imine (PPI) as well as folic acid to fifth-generation polypropylene imine. The conjugated ligands DOX-PPI-FA and PPI-FA have less haemolytic activity, thus more stable and less toxic [45]. Fluorescence studies showed higher cellular uptake by tumor cell of the formulated conjugate ligand. Results of the study revealed that folic-acid-conjugated PPI dendrimers may be a better choice for anticancer drug targeting in the future.

Samuelson et al. have developed translocator protein (TSPO) dendrimer imaging agent with significantly increased targeting and imaging characteristics. The reported study revealed that TSPO can be used as an imaging agent in brain, breast, and ovarian cancer as well as in prostate carcinoma. The main synthesizing material used to produce TSPO dendrimer was 1-(2-chlorophenyl) isoquinoline-3-carboxylic acid (ClPhIQ acid). Hence, TSPO targeted dendrimer is a real-time imaging agent for breast cancer [46].

#### 5.1.3. Liposomes

Liposomes drug delivery system can change the biodistribution and pharmacokinetics of the drug in such a way that it shows overall improvement in the pharmacological properties of chemotherapeutic agents [47–49]. Due to the success achieved by the liposomal-based chemotherapeutic agents in clinical trials, liposomal formulations are currently used for the treatment of the breast cancer like Doxil liposomal preparation [50]. Liposomes consist of lipid bilayer membrane which surrounds the aqueous core. Depending on the solubility of active pharmaceutical ingredient, either it is loaded to lipid membrane or to the hydrophobic core. On the basis of lamellarity and size, liposomes are classified into three: small unilamellar vesicles, large unilamellar vesicles, and multilamellar vesicles [51]. At present, various kinds of cancer drugs have been loaded to this lipid-based system by using different preparation methods. Liposomes are the potential carrier system for anticancer drugs due to the following three pharmacological parameters.

(a) Liposomes provide slow and sustained release. (b) Liposomes are able to reduce cytotoxicity of chemotherapeutic agents by altering the biodistribution of entrapped drug. (c) Liposomes enhance the drug accumulation.

Doxil, a liposomal-based formulation which consists of cholesterol and high phase-transition temperature phospholipid hydrogenated soy phosphatidylcholine (HSPC) gives a stable drug delivery system with enhanced biocompatibility, efficacy and reduced cytotoxic effects [52]. Anthracycline doxorubicin, an active cytotoxic agent, when encapsulated inside the aqueous core of the liposome, significantly shows decrease in the cardiotoxicity [53]. Hence, higher dose of the chemotherapeutic agents can be given to the patient as in the form of liposomal drug delivery system, which can transfer significant amount of the anticancer drug to the desired targeted site.

Shahun et al. had formulated liposomes of doxorubicin (DOX) which is actively targeted to breast cancer by using engineered peptide ligands, P18-4. The effect of the peptide ligand on breast cancer with respect to accumulation cytotoxicity and growth inhibition was studied by varying the molar ratio of P18-4. It was found that the engineered P18-4 peptide can improve the antitumor efficacy by using optimum density [54]. Urbinati et al. had incorporated histone deacetylase inhibitors (HADCi) which belong to class 1 trichostatin and PXD 101 into liposome in large amounts. Phosphatidylcholine, cholesterol, and distearoyl phosphoethanolamine-polyethylene glycol were used to make liposomes and were used in a ratio of 64 : 30 : 6. Liposomes were checked for their toxicity and were measured in MCF-7, T47-D, SKbr 3, and MDA-MB-231 breast cancer cell lines. Formulation made by Urbinati et al. showed improvement in drug accumulation not only in breast cancer but other cancers also get eradicated [55]. Park had prepared pegylated liposome as a suitable drug carrier for doxorubicin. The study revealed the substantial efficacy towards the breast cancer and reduced toxicity of anticancer drug. Pegylated liposomal doxorubicin can be used either in combination with other chemotherapeutics or as monotherapy for breast cancer. Pegylated liposomal formulation can further be used for molecular targeting [56].

Dagar et al. had developed vasoactive intestinal peptide receptors (VIP-R) as a breast cancer targeted imaging with increased pharmacokinetics, biodistribution and with a better imaging ability. VIP-R, a 28-amino-acid mammalian neuropeptide, was attached covalently to the surface of the sterically stabilized liposomes (SSL) which was further encapsulated to a radionuclide (Tc99 m-HMPAO). Presented study revealed that VIP-R is 5 times more expressive in human breast cancer in comparison to other imaging probes. SSL without VIP showed significantly less accumulation than Tc99 m-HMPAO-encapsulated SSL with VIP [57].

#### 5.1.4. Carbon Nanotubes

The allotropes of carbon with a cylindrical nanoshape structure are called carbon nanotubes. Carbon nanotubes belong to the fullerene structure. Representation of the carbon nanotubes is similar to the rolled sheets of graphene rings. A carbon nanotube provides the variety of promising biomedical applications in comparison to other nonmaterials. Carbon nanotubes are more dynamic and are used potentially not only in cancer cell imaging but are also used for drug delivery system. The unique biological and chemical properties, hollow monolithic structure, nanoneedle shape, and the ability of carbon nanotubes to incorporate any functional group make them a suitable carrier system for chemotherapeutic agents. This allows a passive diffusion of carbon nanotubes across the lipid bilayer, or it may attach to the surface of the cell and subsequent endocytosis (engulfing by cells) takes place [58, 59].

Carbon nanotubes can be categorized into two as follows.

1. Single-walled carbon nanotubes (SWCNTs).
2. Multiwalled carbon nanotubes (MWCNTs).

SWCNTs consist of one layer of graphene sheet with diameter of 1-2 nm and length varies from 50 to several hundred nanometers. On the other hand, MWCNTs are multiple layers of SWCNTs which are coaxially arranged with diameter variation of 5 to 100 nm. SWCNTs and MWCNTs have different mechanisms of cell penetration. By using confocal microscopy imaging, it has been observed that SWCNTs have the ability to incorporate inside the cells, whereas MWCNTs are not incorporated into the cells. Size of the carbon nanotube also affects the cellular uptake; due to this, SWCNTs show localized effect in cell and prolonged distribution [60]. Drug can either be loaded into the carbon nanotubes or be attached to the surface of the carbon nanotube. Attachment of anticancer drug can be done by either noncovalent bonding or covalent bonding, which includes electrostatic interactions *π*-*π* stacking and hydrophobic interactions [61]. Research has been done by Wu et al. to deliver an anticancer drug, 10-hydroxyl camptothecin (HCPT), by covalent attachment on the outer surface of the MWCNT. Similarly, succinic anhydride was reacted with HCPT to obtain carboxylic groups on its surface; amino acids were then incorporated to the MWCNTs. Carbon nanotubes coated with HCPT and amino group were functionalized by carboxylic group. This enhances the cell uptake of MWCNTs-HCPT and increased blood circulation with high drug accumulation to the tumor [62]. Liu et al. had conjugated paclitaxel (PTV) to branched polyethylene glycol chain on SWNTs. SWNTs-PTX conjugate exhibited higher drug accumulation, higher bioavailability, and little toxicity. Murine 4T1 breast cancer model shows suppression in tumor growth, enhanced permeation and retention. SWNTs-PTX delivery is the promising treatment for cancer therapy in the future, with higher efficacy and minimum cytotoxic effect [63]. Chen et al. had developed nanocarbon tube by chemical functionalization of SWNTs (f-SWCNTs) with DSPE-PEG-amine. The conjugate bounded to small interfering RNA (SiRNA) was targeting towards breast cancer. Disulfide bond was used for siRNA-mediated gene targeting. Resulting study shows that there is increase in the uptake of SWNTs-SiRNA by 83.55% into the breast carcinoma B-cap-37. Proliferation inhibition was found to be 44.53% for 72 hours in B-cap-37 cells. This novel strategy of chemical functionalization is effective carrier system and is a very advanced or significant therapy for breast cancer in the future [64].

Avti and Sitharaman have developed europium-catalyzed single-walled carbon nanotubes (Eu-SWCNTs) as cellular imaging probe for breast cancer cells. These probes, when excitated at 365 nm and 458 nm wavelengths, showed bright red luminescence. Mechanism of uptake of Eu-SWCNTs is endocytosis, and it was demonstrated in the study that Eu-SWCNTs showed 95%–100% labeling efficiency. The study revealed that Eu-SWCNT is an excellent cellular imaging probe for breast cancer, having excitation value with invisible range [65].

#### 5.1.5. Nanorods

Morphologically the nanorods are nanoscale materials in nanotechnology. Their dimension varies from 1–100 nm and they can be synthesized chemically. Nanorods have a high surface area and are biocompatible, hence, a promising approach for breast cancer. For gold nanorods, because of their special physicochemical properties, they are widely used for imaging, biosensing, photothermal therapy, and for drug delivery system. Inert and nontoxic nature of gold nanorods makes them a suitable nanomedicines carrier system applicable in biomedical field [66, 67]. Different cellular uptake patterns are being followed by the single gold nanoparticle and aggregated gold nanoparticles, and during their uptake these particles interact with the compartments of cellular membrane [68, 69]. Eghtedari et al. had functionalized gold nanorods (GNRs) for *in vivo* targeting to breast cancer which was grown on athymic nude mouse. Herceptin (HER), a monoclonal antibody, was used to functionalize the gold nanorods by molecular recognition of tumor cells of breast along with PEG (polyethylene glycol). Eghtedari et al. revealed the *in vitro* stability study of fabricated herceptin-PEG-gold nanorods in blood and *in vivo* study for breast cancer in nude mice model for breast carcinoma. To achieve a successful targeting to *in vivo* cancer cell, extra engineering efforts are required to make them stable inside the microenvironment of the cancer cells, biocompatible, have prolonged circulation in the blood to reach targeted site, and able to search cancer cells and bind to them. To prolong the circulation time, gold nanoparticles must be protected from reticuloendothelial system, and for this polyethylene glycol (PEG) has shown a promising effect [70].

Connor et al. had studied the cytotoxic effect of gold nanoparticles as noncytotoxic under suitable experiment condition. Small size of nanorods makes them potentially useful for drug delivery and gene therapy, hence, provides drug delivery system with lower cytotoxicity towards normal cell and increased chemotherapeutic efficiency towards abnormal cancer cell [71]. Xiao et al. had developed multifunctional water-soluble gold nanorods (GNRs) as a nanocarrier for tumor targeting. pH-sensitive behaviour of GNRs causes the release of drug, by minimizing the cytotoxic nonspecific systemic distribution of anticancer drug, during circulation inside the human body side by side increasing the efficiency of anticancer drug to targeting tumor [72].

Likewise, zinc oxide nanorods (ZnO) also provide a promising approach in cancer for imaging and drug delivery system for cancer therapy. ZnO nanorods are self-organizing nanomaterials which can be grown on any substance with high quality of crystalline and amorphous properties. This provides ZnO nanorods with large surface area to volume ratio and higher efficiency for photoimaging. Generally, white light is being observed in photonic device and potentially used in photodynamic therapy. Photosensitizers are being taken by cancer cell in photodynamic therapy for cancer followed by exposure to white light [73]. Zhang et al. had fabricated zinc oxide (ZnO) nanorods as a drug carrier for the anticancer drug daunorubicin (DNR) in photodynamic therapy, by using simple one-step solid-state reaction at a normal room temperature in the air. The investigation revealed that the combination of ZnO-nanorods-DNR has induced remarkable decrease in cytotoxicity of anticancer drug and considerable increase in the cancer cell targeting mediated by reactive oxygen species (ROS) in human hepatocarcinoma cells (SMMC-7721 cells) [74]. Kishwar et al. had conjugated developed ZnO nanorods (ZnO-NRs) with protoporphyrin dimethyl ester (PPDME) and used it in the treatment of breast cancer. ZnO nanorods were developed on borosilicate glass capillaries tip by using aqueous chemical growth technique. Developed PPDME-conjugated ZnO-NRs have induced cell localized toxicity indicating potential application in necrosis of breast carcinoma.

Wang et al. have developed multifunctional nanoparticles of gold and pearls consisting of single amine-modified gold nanorod, and Fe_3_O_4_ “pearls” were used to give final touch with the help of carboxyl group. Reported study demonstrated the effectiveness of the gold nanorod in breast cancer photothermal ablation and dual-mode imaging of breast cancer [75].

## 6. Conclusion

Human breast cancer is still an extremely complex and dangerous disease with multiple questions. Nanotechnology is a fast emerging area of science with potential for imaging, monitoring, diagnosing, and delivery of drug to specific targeted tumor cells. Nanoparticles offer the advanced methods of tumor targeting with improved efficacy and decreased toxicity. Many nanoparticle formulations are already in clinical practices. Ongoing efforts by researchers, scientists, and other medical personnel in the field of nanotechnology will consistently produce the new platform for nanoparticles. In the near future, nanotechnology will not only show a greater application in oncology, but the discipline of medicines will also be benefitted.

## Figures and Tables

**Table 1:** Types of nanocarriers for drug delivery.

Sr. no.	System	Structure	Characteristics	Example of compounds	References
1	Polymeric micelles	Hydrophobic core and hydrophilic shell are formed by assembling amphiphilic block copolymer	(a) Efficient carrier system for hydrophilic drug (b) Biodegradable, self-assembling, and biocompatible (c) Potential targeting (d) Functional modification	PEG-b-p(LA-CO-MCG) PEG-B-PCL Curcumin micelles	[1–3]

2	Dendrimers	Synthetic polymer forming nanosized branched structure with repeated units and regular pattern	(a) Uniformity in size, shape, and branch length (b) Tuned pharmacokinetics and biodistribution (c) Increased surface area, increased loading (d) Targeting is achieved	G4 PAMAM-D DOX-PPI-FA	[3, 4]

3	Liposomes	Lipid bilayer memberane forming self assembled closed colloidal structures	(a) Biocompatible (b) Longer duration of circulation (c) Amphiphilic	DOX-p18-4 Pegylated liposomal of doxorubicin	[1, 4]

4	Carbon nanotubes	Benzene ring forming carbon cylindrical structure	(a) Multiple function (b) Chemical modification (c) Water soluble and biocompatible	SWNTs-PTX SWNTs-siRNA	

5	Nanorods	Metals or semiconducting materials forming rod shape structure.	(a) Efficient loading (b) Increased surface area (c) Biocompatible (d) Specific tumor targeting	HER-PEG-GNRs ZnO-NRs-DNR PPDME-ZnO-NRs	[1, 4, 5]

PEG-b-p(LA-CO-MCG)-CISPLATIN, PEG-B-PCL (polyethylene glycol-paclitaxel), G4 PAMAM-D (G4 polyamidoamine dendrimer), DOX-PPI-FA (doxorubicin-polypropylene imine-folic acid), DOX-p18-4 (doxorubicin-peptide ligands), SWNTs-PTX (single-walled carbon nanotubes-paclitaxel), SWNTs- siRNA (single-walled carbon nanotubes-small interfering RNA), HER-PEG-GNRs (herceptin-polyethylene gold nanorods), ZnO-NRs-DNR (zinc oxide- nanorods daunorubicin), PPDME-ZnO-NRs (protoporphyrin dimethyl ester-zinc oxide-nanorods).
